# The Missing Target: Why Industrialized Animal Farming Must Be at the Core of the Climate Agenda

**DOI:** 10.3390/ani15223256

**Published:** 2025-11-10

**Authors:** Jenny L. Mace, Andrew Knight, Fernanda Vieira, Patricia Tatemoto, Mariana Gameiro

**Affiliations:** 1 Centre for Ethics, Philosophy and Public Affairs, University of St Andrews, St Andrews KY16 9AL, UK; 2 Mace Animal Welfare, Dunnock House, 63 Dunnock Road, Dunfermline KY11 8QE, UK; 3 School of Environment and Science, Griffith University, Nathan, QLD 4111, Australia; a.knight@griffith.edu.au; 4 School of Veterinary Medicine, College of Environmental and Life Sciences, Murdoch University, 90 South St., Murdoch, WA 6150, Australia; 5 Faculty of Health and Wellbeing, University of Winchester, Sparkford Road, Winchester SO22 4NR, UK; 6 Sinergia Animal International, Verein Zum Schutz der Tiere, 1180 Vienna, Austria; fvieira@sinergiaanimal.org; 7 Laboratory of Chemical Neuroanatomy, Department of Anatomy, Institute of Biomedical Sciences, Universidade de São Paulo, São Paulo 05508-000, SP, Brazil; patricia.tatemoto@gmail.com; 8 Mighty Earth, 1701 Rhode Island Ave NW, Suite 3-123, Washington, DC 20036, USA; marianaperozzi@gmail.com; 9 Center for Comparative Studies in Sustainability, Health and Welfare, Department of Veterinary Medicine and Animal Health, School of Veterinary Medicine and Animal Science, FMVZ, University of São Paulo, São Paulo 05508-220, SP, Brazil

**Keywords:** animal agriculture, animal farming, climate change, climate crisis, environment, greenhouse gas, methane, CO_2_ equivalents, land use, biodiversity loss

## Abstract

**Simple Summary:**

Ahead of COP30 (the annual Conference of the Parties of the United Nations Framework Convention on Climate Change) in Brazil, this timely article compared the latest literature regarding animal agriculture’s contribution to climate change and broader environmental harm. The findings suggest that, globally, animal agriculture accounts for between 12 and 20% of global greenhouse gas emissions each year. Actual figures are likely to be higher due to measures that are differentially excluded/included between studies. In light of the forecasted failure to meet global commitments to keep warming to an ideal maximum of 1.5 °C, policy makers at COP30 are urged to enact region-specific commitments to reduce production and consumption of animal-sourced foods.

**Abstract:**

Global greenhouse gas reduction targets are applied to many sectors in many countries, as part of the Nationally Determined Contributions mandated within the Paris Agreement (climate). However, industrialized animal farming is typically missed out or deprioritized. This is despite suggestions that excluding this sector would automatically result in global failure to meet 1.5 °C and potentially even 2 °C maximum temperature rise targets, even if fossil fuel use were to immediately cease. To foster further discussion and assessments about the need for such targets in relation to industrialized animal farming, this study collated and analyzed recent studies on the impacts of industrialized animal farming on the environment. Of the 579 items initially retrieved, 47 studies were shortlisted. Over three quarters (*n* = 37, 79%) of the shortlisted studies were unequivocal concerning the significant negative impact industrialized animal farming has had, and continues to have, on climate change and broader environmental concerns—between 12 and 20% of *all annual global* greenhouse gases, and 50%, 32%, and 76% of all *food-originating* eutrophication, soil acidification, and land use, respectively. This all creates immense contributions to biodiversity loss, which itself further aggravates climate change. The remaining studies did *not* assert that industrialized animal farming had an *in*significant impact; however, their findings complicated the picture in one way or another (e.g., suggesting suboptimal measuring methods) or they had flawed methodologies. As a matter of urgency, the present paper recommends that targets for significant reductions in levels of animal production and consumption should be incorporated into discussions and policies for tackling the climate crisis, such as at COP30.

## 1. Introduction

In recent years, significant attention has been afforded to the contribution of food and agriculture to the climate crisis, such as a special report dedicated to land [1], agriculture’s first themed day in 2022 at the 27th annual Conference of the Parties of the United Nations Framework Convention on Climate Change (i.e., COP27), and the signed *Declaration on Sustainable Agriculture, Resilient Food Systems and Climate Action* [2]. Nevertheless, concerns remain regarding the level of attention that these events and institutions allocate to the contributions of industrialized animal farming to the climate crisis, and broader environmental harms. For instance, despite emission reduction targets being forged at COP21 (as part of the Paris Agreement signed by 196 countries) to keep global warming under 2 °C, and ideally not over 1.5 °C, emissions from animal agriculture often remain specifically excluded from, or without reduction targets, within plans, such as Nationally Determined Contributions and the Global Methane Pledge [3]. Additionally, technological solutions are often focused on at the expense of deeper sector transformations aimed at addressing the root causes of problems [4]. Such concerns are increased by suggestions that the 1.5 °C and 2 °C targets remain out of reach without a food system overhaul [5].

COP30 in Brazil is an apt time (albeit, still very overdue) to reconsider the attention given and commitments made to tackling animal agriculture’s contribution to the climate crisis. Brazil is the world’s top exporter of beef [6], and has become the country with the largest cattle population, at nearly 240 million, comfortably surpassing India’s cattle population, which ranks second with almost 195 million cattle [7]. This is important because industrialized cattle farming is considered a particularly significant contributor to climate change [8], and is the leading cause of Amazonian rainforest destruction and degradation in Brazil, alongside soy production for animal feed [9]. The Amazon plays a fundamental role in the Earth’s climate system and ranks as the world’s most biodiverse region [10]. Unfortunately, however, 18% has already been deforested, and a further 17% degraded, with the bulk of this occurring in Brazil [11]. Biodiversity loss is not only a concern in and of itself; it acts as a further accelerant of climate change, and weakens ecosystems, which hinders their resilience and adaptation to climatic changes [9]. Approximately two million indigenous people also live within the Amazon rainforest, and are directly affected [11].

The most recent global update to the Paris Agreement—the Global Stocktake 2023 (part of COP28 in Dubai)—outlined that while there have been improvements to projected warming, the world is still far from the agreed maximum of 1.5 °C of warming [12]. Thus, there is a need for all signatories and subsequent agreements to be more ambitious in the mitigation actions they commit to. While there have been numerous studies modeling or otherwise assessing animal agriculture’s contribution to climate change and broader environmental harm, systematic reviews dedicated exclusively to the topic are scarce, despite wildly different annual estimations having been suggested over recent decades—including up to a high of (though now widely critiqued) 51% [13].

Hence, the aim of this study was to conduct a ‘rapid review’ regarding the research question: What is the contribution of industrialized animal farming to negative environmental impacts? Of particular interest was climate change and biodiversity loss in the Global South. The term ‘Global South’ refers to countries generally considered to have lower economic and industrial development relative to richer countries [14]. The phrase can be considered loosely interchangeable with, and as an updated term for, developing countries and emerging economies.

## 2. Methodology

A ‘rapid review,’ in accordance with current guidance on conducting official Cochrane rapid reviews [15], was chosen for this study for the efficient production of results. In a rapid review, many of the requirements of the traditional PRISMA guidelines [16], which are used for full systematic reviews, are adhered to regarding an explicit and reproducible systematic protocol; however, a rapid review offers flexibility on some items, thereby allowing comprehensive reviews to be more accessible, which may generate insightful and important results that may otherwise not come to fruition. Rapid reviews are particularly useful for informing public policies, as they efficiently synthesize scientific information to support decision-making.

Specific and official recommendations for rapid review protocols (to be known as PRISMA-RR) are forthcoming by PRISMA [17]. In the meantime, there are minimum interim guidelines [18], which this study has followed. The standard requirements of PRISMA that have *not* been implemented for this study included the following: only one researcher reviewed the available literature, only one database was used, and a more selective search string and search parameters were used. To offset the negative impact of restricting the search to one database, a limited Google Scholar search was utilized in addition to artificial intelligence searches by means of Large Language Models (LLMs), such as Perplexity, as outlined below.

### 2.1. Search Protocol

Scopus was used as the primary database for sourcing relevant literature. It is one of the leading scientific databases, which suits the present field of study [19]. After reviewing key titles of papers in the field, the search string used was: TITLE-ABS-KEY (impact OR contribut* OR cause* OR cost OR influenc* OR account* OR stimul* OR aggravate* W/3 (animal OR meat OR factory OR intensive OR industrial*) W/2 (farm* OR agricultur* OR diets OR livestock) W/3 climate OR environment* OR “biodiversity loss” OR greenhouse OR ghg* OR emissions OR “ecosystem degradation”) AND PUBYEAR > 2009 AND PUBYEAR < 2026 AND (LIMIT-TO (LANGUAGE, “English”) OR LIMIT-TO (LANGUAGE, “German”)) AND (LIMIT-TO (DOCTYPE, “ar”) OR LIMIT-TO (DOCTYPE, “re”) OR LIMIT-TO (DOCTYPE, “ch”) OR LIMIT-TO (DOCTYPE, “bk”)). To prioritize high-quality and peer-reviewed results, the search was limited to articles, reviews, books, and book chapters—excluding theses, conference proceedings, and letters. English and German are the operational languages of the lead researcher; hence, studies were limited to these languages. To prioritize the most recent knowledge using the most recent technological advances in climate science, only studies published between 2010 and 2025 were included. The Scopus search was conducted on 1 April 2025.

The Scopus search was complemented by a Google Scholar search carried out on 5 April 2025. Google Scholar was chosen with the intention of capturing studies that an official scientific database might not, as found by Martín-Martín et al. [20]. The search was limited to the first five pages (50 results in total) of 1.89 million results (ordered by relevance) generated by the following search string, which is identical to the one used for Scopus (as above) but tailored to the Boolean operator requirements of Google Scholar: *impact OR contribute OR cause OR cost OR influence OR account OR stimulate OR aggravate AROUND(3) (animal OR meat OR factory OR intensive OR industrial) AROUND(2) (farm OR agriculture OR diets OR livestock) AROUND(3) climate OR environment OR “biodiversity loss” OR greenhouse OR ghg OR emissions OR “ecosystem degradation” (2010–2025)*. Also on 5 April 2025, the same search string was used on Google’s search engine, limiting the results to the last month exclusively. This was performed to capture any ultra-recent publications undergoing indexing delays in both Google Scholar and Scopus. All three pages of results (22 items) were checked, including manually checking any social media posts among the results if they alluded to a recent relevant peer-reviewed publication.

LLMs are chatbots based on deep learning models that enable generative content, iterative conversation, and deep research using all publicly available content online [21]. A penultimate step to gather relevant literature included use of the LLMs Perplexity Deep Research and Gemini Deep Research on 5 April 2025. These two LLMs were utilized as they both scored highly in a recent review of LLM accuracy and efficacy [22] and were both freely available to the first author at the time of writing. Both LLMs were instructed as follows: “*List academic publications from January 2010 to April 2025 that focus on industrialized animal farming’s contribution to climate change and environmental harm. Please do not provide prose-just a list in APA format.*” See Supplementary Material A for the LLM outputs. Two follow-up queries were asked of Perplexity due to the request originally not being fulfilled and then due to an error being identified in its output. Finally, the most recent report from the Food and Agriculture Organization [23] was included due to the authoritative status of this body on climate change and the matching of all other selection criteria outlined below.

### 2.2. Study Screening

Strict inclusion/exclusion criteria had been decided upon pre-screening to minimize biases arising from viewing papers and results. To be shortlisted for inclusion in the analysis, studies needed to:

- Consider industrialized animal farming’s contribution to climate change and/or broader environmental impacts as a core part of its objective (not merely mention it).
- Mention animal agriculture (or an equivalent term) in the title, abstract, or keywords.
- Put a figure on animal agriculture’s (or a part of its) contribution to climate change (or other environmental harm) via an original assessment or systematic review.
- Include a comparative element—comparing animal agriculture with a reduction in animal use, comparing more intensive with less intensive systems, comparing impacts in or from different regions, or comparing impacts from the farming of different species. This was required so that the contribution figures assimilated could be meaningfully interpreted.

**Figure 1:**
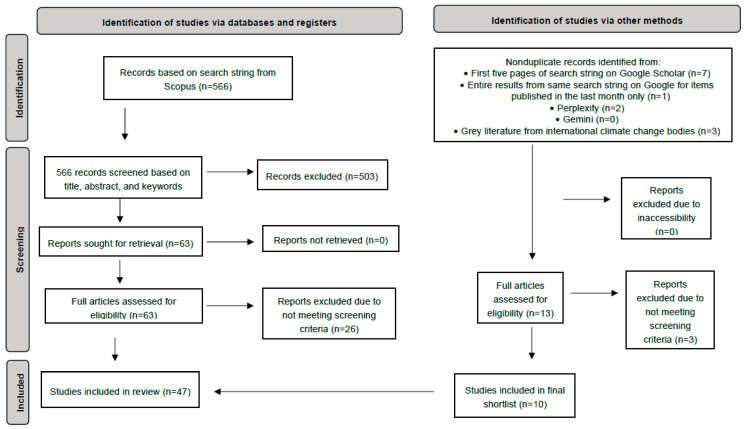
PRISMA flow diagram for systematic reviews, adapted for this rapid review. After Page et al. [16], licensed under CC BY 4.

Studies considering only particular countries, species, animal products, diets, forms of environmental harm (rather than all) were *in*cluded, as were studies of prospective environmental impacts in the future. Studies focusing solely on animal welfare, adaptation/mitigation, arable agriculture, disease risk, novel assessment techniques (unless generating a contribution figure), human health impacts (e.g., from air pollution), or soil quality were excluded. Arriving at a strict definition of industrialized farming is difficult [24]. For the purposes of this paper, we took an *inclusive* approach to defining the term—understanding it to refer to the commercial mass production of animals for food. The main clarity this provided was in the *exclusion* of any studies clearly concerned solely with *non*-industrialized animal agriculture (i.e., smallholdings). Additionally, the majority of review-, argument-, or theory-based items were removed; reviews were only retained that executed some kind of calculation and systematic protocol. A summary of the search and screening protocol can be found in Figure 1.

### 2.3. Study Analysis

Key attributes and results of the shortlisted studies were summarized in a table (Supplementary Material B: Table S1). The analysis comprised simple descriptive statistics and qualitative analysis. Particular attention was given to global findings (stemming from the most comprehensive studies). Particular attention was also afforded to studies focusing on regions of the Global South (the regions forecast to have the biggest growth in animal agriculture), biodiversity loss, and minority viewpoints. Considerable space is given to the minority viewpoint section due to the heavy policy, environmental, and human burden that proposed changes based on a majority viewpoint will have; that is, it is of utmost importance to consider findings as critically as possible and not dismiss contrary voices/findings. A One Health/Welfare lens was adopted for the analysis, which acknowledges overlapping aims of animal welfare, human welfare, and environmental protection [25]. Broom’s [26] definition of sustainability was also adopted, insofar as ‘morality of action’ and animal welfare are included in the understanding of sustainability. Being the most common metric used, greenhouse gas emissions (GHGEs) were particularly focused on for comparative purposes, but other metrics were also considered.

## 3. Results and Discussion

### 3.1. General Characteristics of Shortlisted Studies

An initial 579 items were retrieved from the combined searches; after screening, a total of 47 studies remained for inclusion in this review. Table S1 summarizes the key attributes of the shortlisted studies. They are all journal articles bar item 13, which is a UN FAO report [23]. Nearly 64% (*n* = 30; 63.8%) were published since 2020, with three being published in 2025 and four in 2024. Nine studies (19.1%) had a global focus, while over half (*n* = 27, 57.4%) were based on countries or territories generally considered as developed or wealthy. Ten (21.3%) were based in countries considered either emerging or developing (the Global South); one item (2.1%) focused on one developed and one developing country. All continents featured among the studies bar Antarctica and Africa (apart from in the ‘global’ studies). The shortage of studies focusing on Africa is mirrored in Poore and Nemecek’s study [27]. It may partially reflect the relative dearth of studies in Africa relative to other regions, despite forecasts placing Africa at high risk of climate change impacts, and despite its size and biodiversity hotspots [28]. Nearly three quarters of the studies took a more general meat/reducetarian/plant-based/vegan or broad farmed animal approach (*n* = 34, 72.3%), while 13 (27.7%) focused on one or two particular species or product types (shrimp, sheep, pigs, cattle, red/processed meat, dairy, or chicken/salmon). Studies covering all traditionally farmed animal species, often excluded fish or the farming of other aquatic animals (e.g., [29,30]).

Life cycle analysis (LCA) was the most common methodology used to arrive at the environmental contribution calculations (*n* = 31, 66.0%). Among the LCA studies, several different types of LCA were observed, including regionalized LCA (e.g., [31]), prospective LCA (e.g., [32]), process-based LCA [33], hybrid LCA [34], attributional LCA [35], and both attributional and consequential LCA [36]. It was common for studies to provide insufficient detail regarding the type of LCA employed (e.g., [29]), and the LCA term was used more liberally by some authors than others; some studies examining GHGEs exclusively were named an LCA (e.g., [37]), but others were named inventories (e.g., [38]). LCAs often comprised just one part of a broader method such as concurrent use of GLEAM [39], WEF-based calculations [40], the IPCC’s Tier 1 or 2 [39], an ERF-based DICE model [41], the FoodMIN dietary model [42], or the Nutrition Ecology Framework [43]. Detailed comparisons between these different types of LCA and models are beyond the scope of the present study.

A sizable minority (*n* = 16, 34.0%) relied exclusively on methodologies other than LCAs. These included advanced ERF-based GHG accounting methods [44]; the IPCC’s Tier 2 methodology [8]; network analysis [30]; mass balances [45]; various models/simulations, such as the Monte Carlo simulation [46], optimization linear programming model [47], Dremfia model [48], and CAPRI/MITERRA/GAINS model [49]; inventories [50,51]; reviews [52,53]; or just particular metrics of LCAs such as forms of Global Warming Potential, or GWP (e.g., [54,55]). Data often stemmed from FAO datasheets or other publicly available inventories (e.g., [30,36,56]); or extant meta-analyses (e.g., [57,58]) such as the work of Poore and Nemecek [27], which is also among the shortlisted items within this study.

Cradle-to-farmgate was the typical so-called system boundary applied across all the life-cycle-based studies, but some studies did include at least some post-gate processing [23,27,43,56,59,60,61,62], and even consumption-level assessments such as cooking [35,63]. There were also differences between the studies regarding whether carbon sequestration, indirect/direct land use, and land use change, were included in the assessments.

The environmental metrics that the studies assessed ranged from one—just methane [8,55,64] or just ammonia [45]—to a total of 17 [31]. The terms carbon emissions, GHGEs, or CO_2_ equivalents all typically comprise at least the three main GHGs—CO_2_, methane, and nitrous oxide, which are often all combined into so-called CO_2_ equivalents. For the purpose of distinguishing between focusing on just one gas and multiple gases, the aforementioned terms are regarded as a ‘one+’ metric. As per Table S1, just over half of the shortlisted studies (*n* = 26, 55.3%) focused on either one+ or two metrics; nine (19.1%) focused on three to four; and eight (17.0%) focused on five or more metrics. Collectively, these are the metrics that were used to answer the research question of this study.

Over three quarters of the studies (*n* = 37, 78.7%) indicated that industrialized animal farming was a major causative factor of climate change and broader environmental impact. The 10 studies (21.3%) suggesting a somewhat less significant impact or an otherwise less clear picture are discussed in Section 3.5 below. The nine studies (19.1%) that took a global perspective will be discussed next, followed by a closer examination of the 11 (23.4%) studies that focused on regions of the Global South and/or biodiversity loss. Findings from studies contrasting with the majority viewpoint will then be discussed, as well as further points of disagreement. Studies will be critiqued throughout; the discussion finishes with recommendations and consideration of the present study’s limitations.

### 3.2. Global Studies

The nine studies (19.1%) that conducted a global analysis of industrialized animal farming’s contribution to climate change and broader environmental harm are those by Errickson et al. [41]; FAO [23]; Kuempel et al. [65]; Mendoza [53]; Poore and Nemecek [27]; Wang et al. [30]; Wedderburn-Bisshop [44]; Xu et al. [58]; and Zhang et al. [8]—studies 1, 10, 12–13, 20–21, 23–24, and 35 from Table S1. These all focused on the farming of traditionally farmed animal species, apart from: Kuempel et al. [65], who focused exclusively on farmed salmons and chickens; and Wang et al. [30], who focused on the international trade of livestock, which the authors deemed to contribute 2.5% of all animal agriculture emissions. Zhang et al. [8] focused exclusively on methane emissions and concluded that animal agriculture was responsible for a third of the world’s methane emissions. Mendoza’s [53] paper was appraised poorly due to a lack of key citations, numerous formatting/writing errors, some missing units, missing table column headings, and a lack of clarity regarding some of the logic and calculations used. Thus, this paper will not be discussed further. It is the remaining five studies that will be discussed in more depth.

In three of the remaining five studies, animal agriculture was assessed to be responsible for between 12% [23] and over 20% [58] *of global GHGEs*. The figure stands at approximately 16% according to Poore and Nemecek [27]. From these three studies, Poore and Nemecek [27] were the only authors to study other environmental metrics (e.g., land use, water use) in addition to GHGEs. They found removing meat generated an average of a 76% saving in land use and a 19% reduction in scarcity-weighted freshwater use *in relation to food impacts as a whole*. They were also the only authors to explicitly acknowledge inclusion of aquaculture (farmed fish and crustaceans). They found aquaculture to be responsible for a similar range of methane emissions as ruminants (p. 12) per kilogram of liveweight (but due to manure management rather than enteric fermentation) as well as considerable feed-related emissions ([27]; p. 52, Supplementary Material). The authors summarized that “…meat, aquaculture, eggs, and dairy use ~83% of the world’s farmland and contribute 56–58% of food’s different emissions, despite providing only 37% of our protein and 18% of our calories” (p. 16).

These three studies all shared a similar methodology insofar as they were all based around LCAs. However, differences between their included and excluded criteria were evident. For instance, only Poore and Nemecek included fish farming; only Xu et al. included consumption-level emissions; Xu et al. incorporated a more thorough on-farm carbon flow analysis than Poore and Nemecek; and Xu et al. incorporated changes in the management intensity of land use (not just land use change per se), which is often assumed to be zero. It is understandable that resource limitations and logistical difficulties may lead to the exclusion of farmed fish, of a full account of land use/management, of cooling emissions, of the international trade in farmed animals, of consumption-level emissions, of any post farm-gate emissions, or of (full) carbon sequestration/flow in animal agriculture; nevertheless, for matters of such international significance with important policy implications, there is a huge need for exhaustive and accurate evaluations of animal agriculture’s environmental impacts in future research.

Two final global-oriented studies did not generate standard net CO_2_ and GWP metrics. First, to avoid oversimplification such as overlooking unique properties of different GHGs, Errikson et al. [41] resisted converting non-CO_2_ GHGs to CO_2_ equivalents. They also focused on contributions to temperature change more directly. Under a business-as-usual scenario, they forecast that animal agriculture would be responsible for 0.4 °C (roughly 13%) of the approximately 3 °C of warming predicted by the end of the century.

Second, the study by Wedderburn-Bisshop [44]—the most recent publication of the shortlist—applied novel methods available due to recent advances in climate science. The novel methods centered around consistent gross CO_2_ accounting, emissions-based effective radiative forcing (ERF), global surface air temperature (GSAT) change, and the incorporation of cooling emissions (e.g., when atmospheric pollutants reflect sunlight into space). The author describes how, for instance, it is currently standard for gross carbon to be assessed from fossil fuels, but only net carbon from land use; this is faulty as carbon has an equal likelihood of remaining in the atmosphere regardless of its source, so studies should apply either consistent net or gross carbon accounting across all sectors. ERF and GSAT are less reliant on the operational lifespan of GHGs, meaning methane and CO_2_ can be more readily compared; according to Wedderburn-Bisshop, ERF is now frequently incorporated into aviation climate modeling, which is another sector that has a mix of long- and short-lived emissions (e.g., CO_2_ and methane, respectively).

Rather than assessing animal agriculture’s contribution to GHGEs in one year, Wedderburn-Bisshop’s study determined the proportion of present-day warming that animal agriculture is responsible for from 1750 to 2020 based on ERF, which the author found to be 52% (0.64 °C). This figure includes cooling emissions (usually omitted from fossil fuel calculations despite sulfur and nitrogen (di)oxides having a cooling effect), historical deforestation (as warming from this is still occurring), loss of carbon sink potential resulting from deforestation, and bottom trawling from 1996 to 2020 [66]. The figure is also ultimately based on *conservative* emissions data that exclude fossil fuel use on-/off-farm, all post farm-gate caused warming, agricultural fires, and all fish farming. Agriculture (as a whole) is deemed to be responsible for 60% of present-day warming under the novel method; running the same calculations with the standard GWP metric retrieves a considerably lower estimation of just 33%. The author also finds that the contribution of fossil fuel use to present-day warming under his novel method declines to 18% (from 47% just with the standard GWP metric), highlighting that this is due to accounting for cooling emissions. Wedderburn-Bisshop’s further concerns about accurate methane measures are discussed in Section 3.5. The uniqueness of this study’s approach means that its results cannot be readily compared with those from other extant studies (yet).

The global-oriented studies focusing on animal agriculture generally (versus on particular species) all agreed that ruminants, and particularly cattle, create the biggest impacts, and that land use (change) was the second biggest emissions contributor of the production stage after farm operations, excluding enteric fermentation [23,58]. They all also emphasized the benefits of a significantly reduced intake of animal products on a societal level, especially in richer nations where because meat consumption often remains far higher than in developing countries, the impacts of reducing or eliminating animal product consumption can be all the greater (e.g., [27]; p. 19). The study by Xu et al. may offer the most *exhaustive* GHG estimate of animal agriculture’s contribution to climate change; yet Poore and Nemecek’s [27] study is valuable for inclusion of aquaculture and of four additional metrics, capturing broader environmental harms that are valid in and of themselves but also serve as further fringe contributors to climate change. While Wedderburn-Bisshop’s [44] study is notable for suggesting a new and potentially more accurate approach, more studies using such a method are required for comparative purposes and to include additional parameters that the study excluded, which may further increase animal agriculture’s calculated contributions to climate change. A visual summary of the five most comprehensive global studies can be found in Table 1.

### 3.3. Spotlight on the Global South

The global-oriented studies from the previous subsection also all highlighted regional contributions, both historical and current. For instance, Xu et al. [58] found South and SE Asia to be the only region where plant-based foods have *higher* GHGs than animal-based foods. This is due to high levels of rice production in this region; rice production is an exception to the general trend of plant-based foods being considerably more environmentally friendly than animal-sourced foods, due to high levels of methane emissions. They also found South America to be the largest regional emitter of animal-sourced emissions, with farmland emissions (from grazing and cropland use for feed production), and emissions arising from enteric fermentation and manure management, being the biggest causes. Brazil ranks first both within South America for general emissions *and* out of all countries globally in terms of emissions arising from land use change due to deforestation for animal agriculture (especially for pasture). Brazil, along with Africa, and South Asia, have demonstrated the largest growth in methane emissions over the last decade [8]. The FAO’s [23] report also details how animal production has increased in all country/economy types, with further increases forecast for emerging countries—the largest relative and absolute increases are forecast in Africa and Asia, respectively. It also underscores how, relative to richer nations, emerging countries retain high so-called emission *intensities*, which factors animal productivity into the general emissions. Wang et al. [30] found Africa to have particularly high emissions intensities.

The shortlisted papers also comprised 11 papers that centered on countries in the Global South; the main ones referred to in this discussion are Adhikari and Prapaspongsa [36], Arrieta and González [50], Blanco-Murcia and Ramos-Mejía [43], De Carvalho et al. [54], He et al. [29], Marquardt et al. [32], Marrero et al. [56], and Wei et al. [38]—studies 4, 9, 14, 31–32, 36–37, and 44 in Table S1. The final three applicable studies (studies 6–7 and 46 in Table S1) focused exclusively on either shrimp farming [61,68] or dairy farming [33], and so will only be marginally considered. The eight main studies split into four concerning Latin America/the Caribbean [43,50,54,56] and four concerning Asia [29,32,36,38]. In short, all studies examining the contribution of different foodstuffs found animal products to have the biggest environmental impact; Wei et al. [38] did not focus on such comparisons, but noted legislation surrounding targeted sector GHGE reductions did actually result in GHGE reductions.

The most comprehensive of the aforementioned Latin American/Caribbean (LAC) studies is by Marrero et al. [56], which focused on the LAC region as a whole. The authors found that, despite meat comprising 7.5–12.7% of these populations’ food consumption, animal-sourced foods accounted for 85.3% of *the region’s total food emissions* for South America, 87.1% for Central America, and 43.8% for the Caribbean (with meat accounting for the bulk of these figures). South America and the Central America’s figures are substantially higher than the *global average* of 57% reported by Xu et al. [58], while the Caribbean’s is less than the global average, at 43.8%. The paper does not provide details on the LAC’s total food emissions and also uses different CO_2_ equivalent units (that are not accurately convertible) than the global studies papers discussed above; thus, a figure for the contribution of animal-sourced food relative to LAC’s *total GHGEs* (not just food GHGEs) cannot be provided. Animal sourced foods were also found to contribute up to 56.6% of *total food-based* land use, and 54.2% of *total food-based* water consumption. In contrast, the authors found both local and imported plant-based food items to have the lowest environmental impact across the same three metrics. The authors described the LAC’s unexpected pairing of farm intensification and a 10% increase in food insecurity and malnutrition over five years. Interestingly, legumes and seeds were found to be particularly low impact, especially relative to nutrition content.

The most comprehensive of the aforementioned Asia-focused studies is by Adhikari and Prapaspongsa [36]. It featured Saudi Arabia, India, Thailand, China, and Japan (the latter not generally considered a developing country). It assessed six environmental metrics—GWP, terrestrial acidification, eutrophication, eco-toxicity, human toxicity, and fossil resource scarcity—of different food types using a cradle-to-farmgate LCA method. This study was the only shortlisted item to explicitly state inclusion of both ALCA and CLCA methods; the former focuses more on environmental factors that a product is responsible for, while the latter focuses on how changes to a product may affect environmental factors [69]. Taking into consideration the proportion of different food types in each population’s diet, on a per kilogram basis, it found that in all countries bar India (due to low meat consumption), meat was the highest contributor to environmental harm, except for the eutrophication metric. When modeling general calorie reduction (for all countries bar India as India was not found to be overconsuming calories on a per capita basis), as well as animal product reduction and cereal reduction, it also found that the animal product reduction diet scenario achieved the greatest reduction in environmental impacts in all countries bar China (39%, 36%, and 31%, for Thailand, Saudi Arabia, and Japan, respectively). The study also suggested that had there not been errors in the vegetable data used for China (overestimations), then successful impact reductions would also have been seen for China. The same result trends are seen across both the ALCA and CLCA methods.

These studies demonstrate that benefits associated with reductions in animal product consumption are not restricted to the future for many countries in the Global South; current consumption levels are already too high in terms of environmental and health impacts. Additionally, care must be taken with blanket application of calorie reduction plans, as some emerging economies have not yet reached caloric sufficiency.

### 3.4. Spotlight on Biodiversity Loss

Four of the shortlisted studies incorporated specific measures of biodiversity impact in their studies [32,51,52,70]. These studies all focused on Europe, although Marquardt et al. [32] focused on both Germany and Indonesia. The studies all found considerable biodiversity impacts from animal agriculture. It accounts for 78% of overall agriculture’s terrestrial biodiversity loss (which stands at 38%) according to Leip et al. [70], while Head et al. [51] and Marquardt et al. [32] both found that their respective vegetarian ‘meat’ and plant-forward meal options had significantly lower biodiversity impacts (especially in Germany versus Indonesia). For instance, Germany’s current meat-based biodiversity footprint was found to be roughly 3.5 times that of the plant-based food.

The main cause of the biodiversity losses captured in the aforementioned studies was land use (transformations) resulting from pasture and feed production. As Leip et al. [70] and Head et al. [51] explain, land use is a multifaceted metric. It can cover use of land for pasture or for growing feed crops; land that was long ago transformed from its original vegetation, or land that was more recently or currently changed for its present purpose; land use that is managed to different intensities; and land in different regions that has different degrees of contributions to biodiversity. Yet another nuance that should be addressed in future studies is indirect conversions of virgin forest via purchasing of pasture land, which can lead to former pasture owners using the funds to buy up larger swathes of cheaper virgin forest. Indeed, according to Berengeur et al. [9], the proliferation of soy crops in the Brazilian Amazon is occurring mostly on such pasture land.

There are other relevant metrics for biodiversity losses too—namely, eutrophication and acidification of waters, stemming largely from nitrogen-based emissions from manure and fertilizer use on feed crops—as these all change the ecosystems and so impact the fauna present [70]. Thus, even if direct measures of a biodiversity score are absent, many studies are relevant due to inclusion of metrics such as land use, eutrophication, and acidification. Poore and Nemecek [27] are the only authors among the global oriented studies to have focused on these broader parameters. They found the least impactful animal-sourced foods still emitted more eutrophying and acidifying emissions than plant-sourced food (except nuts); they found a global shift to animal-free foods could reduce both acidification and eutrophication *from all food* by approximately 50%. Considering that food agriculture is currently responsible for 78% of eutrophication and 32% of terrestrial (soil) acidification, this is hugely significant. Other shortlisted studies confirmed these significant reductions in eutrophication and acidification with shifts toward more animal-free foods (e.g., [27,31,36,49]). Ultimately, GHGEs also affect biodiversity, meaning all metrics discussed above are relevant to biodiversity. This highlights the positive feedback loop of biodiversity loss: it is both a contributor to, and consequence of, environmental harm and climate change.

### 3.5. Disagreements Among the Studies

As mentioned, 10 studies (21.3%) indicated a more equivocal impact from industrialized animal farming on climate change, at least regarding particular aspects of it. Five of these studies found that a *lower* intensity of farming resulted in *higher* GHGEs, spanning dairy cattle in China [33], shrimp farming in Indonesia [68], dairy sheep in Spain [71], dairy goats in Greece [47], and farming of multiple species in China [38]. A further study regarding sheep meat in Ontario (Canada) found that farming intensity was unimportant in terms of increasing/decreasing GHGEs [72]. However, it is worth noting that there are studies from the shortlist claiming the opposite, including from the same continent and studying the same species [61]. Moreover, while it is true that less intensive farming can pose *more* environmental problems in relation to some metrics (e.g., direct land use), this constitutes an *additional* reason for transitioning away from industrialized animal agriculture, rather than a reason for attempting sustainable intensification. This is for two reasons: (1) While some studies suggest some level of reduced GHGEs from higher intensity animal farming, this is relative to a lower intensity of animal farming—*not* to arable farming, for which the savings would be much more significant as discussed throughout this paper; (2) From a One Health/Welfare lens, supposed sustainable intensification is actually a contradiction in terms due to significant animal welfare compromises and increased zoonoses risks (e.g., see [26,73]). Particularly alarming is the concept of *super*-intensive animal farming, introduced by Tamariska et al. [68].

Two other studies (among these counter studies) focused on dietary shifts: a 20% reduction in red meat in Finnish diets [48] and the removal of meat from citizens’ diets in the USA [74]. Lehtonen et al. found that a 20% reduction in exclusively red meat from Finnish diets neither lead to significant reductions in GHGEs nor in land use or total farmed animal production. This was due to any benefits being countered by increases in consumption of white meat, fish, dairy, and eggs [48]. Again, far from being a justification for *not* pursuing reductions in animal production and consumption, this study actually points to the need for *greater* reductions than 20%. Other dietary scenarios studied in the shortlisted studies retrieved by the present study, as a minimum incorporated a 50% reduction, but commonly 70–100% plant-based scenarios too. These consistently demonstrated superior results in terms of significantly reduced environmental impacts (e.g., [32,37,40,42]). The Lehtonen et al. [48] study demonstrates the need for reductions to span across meat and animal products from *all* farmed animal species rather than exclusively focusing on red meat—despite the majority of GHGEs, land use, and water use stemming from ruminant animals. This would also improve animal welfare across farmed species more broadly, and reduce disease risks too [73].

The second of the aforementioned studies, by White and Hall [74], found that removing all animals from agriculture in the USA only contributed to a 28% reduction in GHGEs *from overall agriculture*—amounting to only a 2.6% reduction *from total US GHGEs*, which is substantially less than the global averages discussed in Section 3.2 above. However, some significant assumptions were made in this study, as follows. First, the study assumed that a portion of crops grown would need to be fed to the population of cats and dogs in the US; however, this may not be necessary in the future, given recent developments in, and the first sales of, cultivated meat-based pet food and pet food based on proteins from microbial fermentation [75,76]. Second, byproducts from crops need not be disposed of as the authors assume, but could alternatively be recycled into pet food or energy creation [77].

White and Hall also found that removing animal agriculture contributed to an increase in nutritional deficiencies; however, the proposed nutrient deficiencies are effectively unfounded as the study rested on the following incorrect assumptions: (1) There were minimal changes within the simulated model regarding crops grown to safeguard human health, resting on the incorrect assumption that humans would eat the same as the prior livestock herd. The proportion of vegetables and fruits grown actually even *reduced* in quantity in the simulated animal-free models (p. E10304-5). (2) Zero national/personal nutrient supplementation. The most essential nutrients such as B12 can be supplemented at the personal or even national level (akin to iodized salt in many countries, including Brazil). Indeed, several other of the shortlisted studies found nutritional *sufficiency* and benefits associated with a 75–100% animal-free diet (e.g., [35,56,62]).

A final two studies focused on perceived inaccuracies in conventional measurements of methane emissions when using GWP100, which is the standard metric used in most studies [55,64]. The authors’ concerns lie in GWP100’s tendency to *over*estimate impact measurements from *declining* methane emissions, while *under*estimating the impacts from *rising* methane emissions. These authors instead advocate using GWP STAR (GWP*), which factors in the short-lived nature of methane emissions, relative to CO_2_. Using GWP*, Liu et al. [55] found that California’s dairy farming methane emissions would reach neutrality within 10 years if a 1% annual reduction could be achieved, while Pressman et al. [64] echoed this trend. Wedderburn-Bisshop [44], whose paper is discussed in Section 3.2, shares these authors’ concerns about accurate methane impact measurements; however, he argues that the 100-year timespan applied to any GWP model is random—as Pressman et al. themselves also state (they also apply it to their GWP*).

Wedderburn-Bisshop instead favors a much longer timespan, affirming that methane has no cooling co-emissions and remains a key contributor to present-day warming. He also suggests that his novel method could help to even out the playing field in terms of historical and current emissions as it means past deforestation in the West is included, potentially creating more fairness between the Global North and South in mitigating climate change. It is also likely that Pressman et al. and Liu et al.’s studies are very region-specific; they are focused on declining dairy cattle populations in the USA, while global livestock populations are increasing for the foreseeable future. In summary, Liu et al. [55] and Pressman et al. [64] found that methane emissions are *over*estimated in standard GWP metrics, while Wedderburn-Bisshop found that methane emissions are *under*estimated in standard GWP metrics. Unfortunately, the respective papers do not critique the other proposed more advanced approach in each other’s papers—only the more standard GWP metric. Determining and finding the most accurate methane emission measurements is an on-going and evolving area of research with GWP* quite a new method and the collection of novel approaches advocated by Wedderburn-Bisshop even newer. Thus, future research should test the authors’ findings and claims further. In the meantime, a multitude of environmental problems caused by industrialized animal farming remains, aside from methane emissions.

### 3.6. Recommendations

In recognition of the findings from this study and the predicted global failure to meet the Paris Agreement targets [12], we strongly urge global commitments to reduce reliance on industrialized animal agriculture and to shift toward more plant-based diets as a matter of urgency, in order to minimize global warming, environmental degradation, and biodiversity loss. Such commitments need to be tailored to regional unique needs, and also need to recognize the roles some emerging food technologies can make. It will be important to emphasize the accompanying personal health, public health, and cultural benefits (e.g., re-connection with indigenous traditions) that can arise from such shifts, alongside the environmental and welfare benefits, and to apply principles of a ‘just transition’ [78].

It is difficult to arrive at a target percentage reduction in animal-based foods, but *the higher, the better*. Such targets should avoid singling out particular farmed animal species or meat types. Any animal farming that is retained should become less intensive with GHGE mitigation measures incorporated; lowering emissions intensities in the Global South through animal health/welfare measures may offer low-hanging fruit in this regard while increasing food security. Rice cultivation methods should also be refined in terms of lowering methane emissions.

Research should also continue, especially in regard to determining the most accurate methodologies for arriving at environmental impacts and in regard to closing the research gap pertaining to climate research in Africa. Finally, the most accurate figures regarding animal agriculture’s contribution toward climate change, would only increase with the inclusion of farmed fish and wild-caught fish—future studies should definitely include these estimates. Additionally, trillions of insects are now farmed annually [79]; future studies should also begin to include insect farming. Determining the most accurate sector contributions is important for effective mitigation and adaptation policies.

### 3.7. Limitations of the Current Study

The present study followed a systematic method to minimize bias, with sufficient detail stipulated to allow replication. Nevertheless, due to being a rapid review rather than a full systematic review, there is an increased chance of relevant studies having been missed in this review through use of a more selective search string and only one search database. It is particularly evident that the review did not pick up on any studies situated in Africa. The risk of missing a representative sample of studies was minimized by checking the first five pages of results from the same equivalent search string used on Google Scholar. Shortlisted studies included studies with contrasting (as well as similar) results; thus, there can be high confidence in bias minimization in this regard.

Relatedly, of note is that the guidelines for conducting rapid reviews stem from the biomedical field [15], which may affect the suitability of the method for non-medical fields. However, the same is true of the PRISMA guidelines for systematic reviews [16], yet these are successfully and commonly applied across different disciplines. Most significantly, the guidelines for rapid reviews clarify how rapid reviews are suited for timely decision-making, fast-evolving fields, and multiple stakeholders, which complements the purpose of the present study [15]. Additionally, while the strengths and weaknesses of the included studies were critically considered, a thorough and reproducible quality appraisal of each item was not performed. However, only one out of 47 studies could not be meaningfully discussed due to poor write-up quality, and one other was found to have a particularly poor methodology; the former of these studies was strongly opposed to industrialized animal agriculture and the latter strongly opposed to a plant-based food transition.

It should also be noted that the lack of content regarding differential post-farmgate (e.g., forms of product processing) and consumption-level (e.g., cooking) environmental impacts is due to the predominant use of the ‘cradle-to-farmgate’ timespan within lifecycle analyses [80]. Similarly, the overrepresentation of ruminant species (and products from them) reflects the greater attention afforded to these in the literature and, generally speaking, their larger environmental impacts relative to those of other species. Due to finite resources for this project, a meta-analysis was not within its scope; however, this is to be encouraged as a next step to optimize comparability. Finally, it was not always possible to disentangle industrialized animal farming from non-industrialized animal farming. For instance, some studies did not define farm types and some included multiple farm types. Due to the difficulty in defining industrialized animal farming, we tried to minimize the impact of this by taking an inclusive approach and through liberal use of this phrase—only excluding studies that explicitly focused on small holdings.

## 4. Conclusions

Over three quarters of the 47 shortlisted studies (among 579 initially retrieved items) affirm a clear-cut significant negative impact of industrialized animal farming on climate change and broader environmental aspects. In regard to greenhouse gases specifically, at the top-end of estimations, animal agriculture is responsible for at least 20% of global greenhouse gas emissions annually, and as much as 52% of present-day warming, with methane emissions being particularly culpable. Animal agriculture also comprises over 80% of all farmland worldwide, despite only providing 37% of protein and 18% of calories worldwide. In light of this, specific greenhouse gas reduction targets for industrialized animal agriculture—especially for methane emissions—must be considered in public policies and global agreements. Additionally, it is important to look beyond greenhouse gas emissions for the fullest account of climate change; biodiversity impacts via land use (change), eutrophication, and acidification, must also be factored in. It is imperative that collective scientific evidence is taken into consideration and that new agreements lead to the effective implementation of policies worldwide.

## Figures and Tables

**Table 1:** Key attributes and findings from the most comprehensive global studies included in this review.

Study	Contribution of Animal Agriculture to Global GHGEs	Alternate Primary Metric	Key Parameters Included
Xu et al. [58]	20%	N/A	- Consumption-level emissions - Thorough on farm carbon flow - Changes in management intensity of land use
Poore & Nemecek [27,67]	16%	N/A	- Aquaculture - Some post-farm processing (e.g., transportation) - Separate figures for broader environmental impacts (land use, water use, eutrophication, acidification)
FAO [23]	12%	N/A	- Some post-farm processing (e.g., transportation)
Wedderburn-Bisshop [44,66]	N/A	52% (proportion of present-day warming animal agriculture is responsible for)	- Bottom-trawling - Cooling emissions - Historical deforestation
Errickson et al. [41]	N/A	13% (proportion of business-as-usual 3 °C warming by the end of century)	- Some post-farm processing (e.g., transportation)

Note: The studies are listed both by metric category and by descending order of contribution to GHGEs. Not all included parameters are listed—only those particularly of note (as they are usually excluded). Additionally, unless otherwise stated, impacts from fisheries, aquaculture, insect farming, and post-farmgate processes were excluded from studies, though Wedderburn-Bisshop [44] did include one impact from fisheries—bottom trawling. ‘N/A’ pertains to no measure of certain metrics in the studies concerned.

## Data Availability

Data sharing is not applicable.

## Supplementary Materials

The following supporting information can be downloaded at: https://www.mdpi.com/article/10.3390/ani15223256/s1, (A) LLM Outputs, (B) Table S1: A summary of the 47 shortlisted studies. References [81,82] are cited in the Supplementary Materials.
