# Supplementary material for: The Missing Target: Why Industrialized Animal Farming Must Be at the Core of the Climate Agenda
Source: Animals (Basel). 2025 Nov 10;15(22):3256. doi: 10.3390/ani15223256 (PMC12649370; doi:10.3390/ani15223256)
Supplement: Supplementary file 1 [file animals-15-03256-s001.zip › Supplementary Material B - Table S1.pdf]

Supplementary Material B

Table S1. A summary of the 47 shortlisted studies, ordered by publication date.

Note: Red boxes denote studies that are in the minority insofar as the studies in some way de-problematize industrialized animal farming regarding environmental impacts to lesser or greater degrees. Individual environmental metrics are not listed if numbering five or more; the number of metrics investigated is just stated. Abbreviations: LCI = life cycle inventory; LCA = life cycle analysis; PLCA = prospective LCA; ALCA = attributional LCA; CLCA = consequential LCA; RLCA = regionalized LCA; LCSA = life cycle sustainability assessment; GHGA = greenhouse gas accounting; ERF = effective radiative forcing; GHG = greenhouse gas(es); GHGEs = greenhouse gas emissions; ADP = abiotic depletion potential; GWP = global warming potential; GWP* = GWP Star; SLCP = short-lived climate pollutants; MTP = marine aquatic ecotoxicity potential; AP = acidification potential; EP = eutrophication potential; WEF = water, energy, food; ED = non-renewable energy demand; WD = water depletion.

	Author	Year [citation]	Shortened title	Country / region	Species / food type	Study type	Environmental metric assessed	Key results / contribution figure(s)
1	Wedderburn-Bisshop	2025a [44]	Increased transparency in accounting conventions could benefit climate policy	Global	General animal agriculture	Advanced ERF-based GHGA	Carbon emissions, land use	"Updated and advanced GHG accounting methods demonstrate:<br>- Increased carbon emissions from deforestation.<br>- Fossil fuels are responsible for 18% of ERF, a reduced contribution due to masking from cooling co-emissions.<br>- Agriculture as the leading cause of present-day global warming, mainly due to CO_2 from past and present deforestation and methane.<br>- Unlike fossil fuels, agriculture has produced minimal cooling emissions to mask its' warming.<br>- Agriculture provides 'low hanging fruit' mitigation opportunities.<br>- GWP100 understates present-day warming from methane."
2	Saarinen et al.	2025 [37]	Dietary climate impact correlates ambiguously with health biomarkers	Finland	Meat vs less/no meat	GWP-based LCA	CO_2 equivalents	"The climate impact (CO_2 eq.) for PLANT was 3.32 kg per day, 3.05 kg per 2,000 kcal, and 0.04 kg per gram of protein, for 50/50 4.34, 4.20, and 0.05 kg, and for ANIMAL 4.93, 4.94, and 0.06 kg, respectively ($p < 0.05$ for all except ANIMAL vs. 50/50 /g protein and /2,000 kcal). Replacing animal-sourced proteins with plant-sourced proteins reduced the climate impact of the diet."
3	Deteix et al.	2025 [31]	Joint assessment of the environmental impacts and resource criticality of French food consumption scenarios	France	Five diet / agriculture scenarios	RLCA	17 aspects	"Three of the four scenarios have lower environmental impacts than the BAU scenario for all impact categories. For climate change, impacts could be reduced by between 25 % and 45 %. Similar results were observed for resource criticality. These results were mainly explained by the change in diets, with lower levels of overall consumption, and less animal products in proportion. Regionalisation of impacts can lead to variations of up to 50 %."

4	Marq-uardt et al.	2024 [32]	Prospective life cycle assessment of climate and biodiversity impacts of meat-based and plant-forward meals	Indon-esia / Ger-many	Meat-based / plant-forward meals	PLCA / LCI	Climate footprint / biodiversity loss	Germany: "[T]he German meat-based meal (beef bolognese) consistently has the highest impact, while the German plant-forward meal (lentil bolognese) consistently has the lowest ... The largest reduction in impact is seen in the most ambitious [green route] (SSP1) scenario (an 18% reduction in climate impact and 29% reduction in biodiversity impact)." Indonesia: "[T]he absolute difference in impact between the baseline plant-forward and meat-based meals is smaller than that seen in Germany. In addition, for both metrics, when the impacts are considered per serving the meat-based variant has the higher impact, however when considered per 100 kcal the plant-forward variant has the higher impact. The climate and biodiversity footprints for both Indonesian composite meals are lower than the baseline across all three future scenarios and are consistently lowest in SSP1 ... "
5	Góralska-Walczak et al.	2024 [57]	Environmental indicators of vegan and vegetarian diets	Poland	Veg* vs meat diets	Footprint LCA data from Poore & Nemecek (2018)	Land use, carbon footprint, water footprint	"Studied vegetarian and vegan diets were characterized by 47.0% and 64.4% lower carbon footprint, 32.2% and 60.9% lower land use indicators, and 37.1% and 62.9% lower water footprints, respectively, compared to the meat-containing diet. Animal-derived foods, including milk and dairy, appeared to be the main contributors to all three environmental footprint indicators of both the meat-containing and the vegetarian diets."
6	Han et al.	2024 [33]	Industrialization mitigates greenhouse gas in China's dairy sector	China	Dairy	GWP-based process-based LCA (imported feed excl.)	GHGEs	"Industrial systems exhibited lower methane but higher carbon dioxide intensities, with net GHG intensity lower than other systems." "[Industrialization] may prove insufficient to offset emissions from future production expansion."
7	Tamar-iska et al.	2024 [68]	Towards sustainable shrimp farming: life cycle assessment of farming practices	Indon-esia	Shrimp farming	GWP-based LCA (cradle-gate)	5 aspects	"[I]ntensive shrimp farming results in an 11.21% higher in ADP, a 52.22% higher in GWP, a 73.70% higher in MTP, a 42.11% higher in AP, and a 65.13% higher in EP compared to super intensive farming ... super intensive farming system has the potential to result in a reduced potential environmental impact."
8	Press-man et al.	2023 [64]	Methane emissions from California dairies estimated using novel climate metric show improved agreement with modeled warming dynamics	Cali-fornia, USA	Dairy cattle	SLCP-based GWP* (not cradle-to-gate, just trial)	Methane	"Average CO_2 warming equivalent emissions given by GWP* were greater than those given by GWP under increasing annual CH_4 emissions rates, but were lower under decreasing CH_4 emissions rates ... [C]umulative CO_2 warming equivalent emissions given by GWP* matched modeled warming driven by decreasing CH_4 emissions more accurately than those given by GWP ... GWP* represents methane's short-lived nature ... its ability to unambiguously link warming impacts to SLCP emissions, GWP* may provide a more accurate tool for quantifying SLCP emissions into policy contexts."

**9**	He et al.	2023 [29]	Study on the relationship between economic growth of animal husbandry and carbon emission	China	Beef cattle, cows, pigs, poultry	LCA (type not specified)	Converted carbon emission equivalents	"From 2000 to 2020, the carbon emissions of animal husbandry in Heilongjiang Province showed an overall slightly upward trend. From the perspective of various emission links, the highest carbon emissions are from the gastrointestinal fermentation environment (42.49%). The main driving force behind the continuous increase in carbon emissions from animal husbandry in Heilongjiang Province is the changing factors of agricultural population returns and changes in the production structure of animal husbandry."
**10**	Mend-oza	2023 [53]	Transforming meat based to plant based diet is addressing food security and climate crisis in this millennium: a review	Global	Meat vs plant	Review	CO_2eq	"Our calculation showed that transforming meat based diet to plant based diet, the avoided emissions is 22.681 billion tons CO_2eq (72%). While beef consumed is only 59.1 million tons, the carbon emissions per ton of beef is 221.63 ton CO_2eq, hence the highest at 13.098 billion tons CO_2eq.(42%) of all meat … A 50% carbon emissions reduction by 2030 can be achieved by reducing meat consumption by 50% +50% green production. Net zero emissions by 2050 can be achieved by 100% Green Production and 80% less meat …"
**11**	Jenn-ings et al.	2023 [81]	Five U.S. Dietary patterns and their relationship to land use, water use, and GHGEs	USA	Five dietary patterns	Secondary LCA-based data (types not specified)	Land use, water use, GHGEs	"Results indicate that the three omnivore diets contributed the greatest to land use, water use, and GHGEs [current diets: 5.17 m^2 per day, ~430L/day, 3.19 CO_2 eq/day, respectively]. The Vegan diet scored the lowest across all indicators [1.82 m^2, 404L/day, 0.72 CO_2 eq/day, respectively], although the water required for plant-based protein nearly offset other water gains. For the omnivore diets, red meat and dairy milk contributed the most to each environmental indicator."
**12**	Kuem-pel et al.	2023 [65]	Environmental footprints of farmed chicken and salmon bridge the land and sea	Global	Chicken, salmon	Adapted LCA (partial, incl. geographic)	Cumulative GHGEs, nutrient pollution, freshwater use, spatial disturbance	"The footprints of both sectors are extensive, but 95% of cumulative pressures are concentrated into <5% of total area. Surprisingly, the location of these pressures is similar (85.5% spatial overlap between chicken and salmon pressures), primarily due to shared feed ingredients. Farming broiler chickens disturbs 9 times more area than farming salmon (924,000 vs. 103,500 km^2) but yields 55 times greater production. Environmental pressures from feed ingredients account for >78% and >69% of cumulative pressures of broiler chicken and farmed salmon production, respectively."
**13**	FAO	2023 [23]	Pathways towards lower emissions: livestock agrifood systems	Global	All main land (6)	GLEAM, Tier 2 LCA, direct/ indirect, cradle-to-packaging	GHGEs	"For the reference year of 2015, livestock systems with cattle, buffaloes, sheep, goats, pigs and chickens collectively contribute to 6.2 Gt CO_2eq emissions, constituting approximately 12 percent of all anthropogenic GHG emissions. It is important to note that this figure is lower than previous GLEAM estimates, but direct comparisons are not feasible due to differences in methodology, input data and global warming potential (GWP) values."

14	Marr-ero et al.	2022 [56]	An integrated assessment of environmental sustainability and nutrient availability of food consumption patterns	Latin Am-erica, Cari-bbean	General animal agricult-ure	Region-specific LCA (cradle-to-slaughter?)	GHGEs, land use, freshwater consump-tion	"Annual overall GHGEs were highest in Caribbean food consumption patterns (2521.2 kg/capita), largely derived from meat (26.7%) and fruit (23.8%) production. Land use (1941.0 m^2/capita) and water consumption (2060.8 m3/capita) ... were highest for the South American pattern, owing to high consumption of meat and dairy. Across the regions, meat constituted 7.5%–12.7% of food consumption yet accounted for as much as 73.1% of GHGEs, 56.6% of land use, and 54.2% of water consumption ... legumes and seeds, cereals, roots, and vegetables demonstrated ... low environmental impacts, particularly relative to ... dietary protein, iron, and zinc."
15	Allen-den et al.	2022 [40]	What should we eat? Realistic solutions for reducing our food footprint	High-income coun-tries	Plant-rich diets	Review: use of LCA- and WEF-based calcula-tions	Impact score: GHGEs, land, water	"Compared with the baseline omnivore diet, all diets had lower GHG emissions (median −11% to −48%) and land-use (−10% to −44%), with the vegan diet having the greatest median reduction. All diets used less water than the baseline omnivore diet (−6% to−37%), with the exception of the vegan diet (+1.5%)."
16	Akam-ati et al.	2022 [39]	Comparative assessment of greenhouse gas emissions in pig farming	Europe	Pig (semi-intensive vs intensive)	Tier 1 & 2 (IPCC), GLEAMi (FAO)	GHGs: CH_4, direct/indir-ect N	"The semi-extensive farm had lower emissions/fewer animal compared to the average intensive pig farm in the Greek territory. The Tier 1 approach revealed that breeding animals produces more to the emissions, contrary to Tier 2, which showed that fattening pigs is responsible for the majority of GHG emissions."
17	Bhatt et al.	2022 [72]	Life cycle impacts of sheep sector	Ontario, Canada	Sheep (intensity consid-ered)	LCA (cradle-to-farm gate)	Global warming, ED, WD	"Enteric emissions from livestock are responsible for 39% of greenhouse gas (GHG) emissions, followed by feed production (29%), farm operations (23%), and manure management (10%). ED and WD impacts are each roughly split evenly between feed production and farm operations ... farming intensity does not have a significant effect on impact scores."
18	Hendrie et al.	2022 [34]	Comparison of current diets with the Australian dietary guidelines (ADG) and the eat-lancet planetary health diet (PHD)	Austr-alia	Meat, dairy, alterna-tives	Secondary survey and data from prior hybrid/process-based LCA studies	Environmental impact score: climate/ water / crop scarcity footprints	"The environmental impact scores of the PHD and ADG were 31% and 46% lower than the average Australian diet (AAD). The AAD contained two to almost four times the ADG and PHD maximum recommended intake of discretionary choices, and provided inadequate amounts of the vegetables, cereals, unsaturated fats and meats and alternatives food groups. [It] also contained less dairy and alternatives than the Australian Dietary Guidelines. In the AAD, red meat and poultry contributed 73% to the total servings of meat and alternatives compared to 33% and 10% for the ADG and PHD respectively."
19	Ruett et al.	2022 [46]	Accounting for uncertainty of life cycle assessments	Europe	Meat vs less / no meat	Monte Carlo simulation	Carbon footprint	"The range and absolute value of a diet carbon footprint become larger the higher the amount of products with highly varying emission values in the diet. All dietary pattern carbon footprints overshoot the 1.5 degrees

			by applying a probabilistic approach			(un-certainty analysis)		threshold. The vegan, vegetarian, and diet with low animal-based food intake were predominantly below the 2 degrees threshold.”
**20**	Wang et al.	2022 [30]	The spatiotemporal patterns and network characteristics of emissions embodied in the international trade of livestock products	Global	Meat of cattle, sheep, goat, pig, chicken; milk, eggs	FAOSTAT Emission Intensities / Network analysis	GHGs	“Total volume of GHG emissions embodied in livestock product trade reached 92.0 MT in 2017, accounting for 2.6% of the total emissions from livestock production. Sheep meat has replaced cattle meat as the major contributor to embodied emissions. In 2017, the largest flows of embodied emissions were within Europe, followed by the flows from Oceania to Asia … a significant increase was witnessed in goat meat and chicken meat trade-related emissions. The global increases in the transfer of GHG emissions embodied in livestock products globally were lower than the increases in the volume of livestock trade.”
**21**	Zhang et al.	2022 [8]	A 130-year global inventory of methane emissions from livestock	Global	General animal agricult-ure	Tier 2 (IPCC)	Methane emissions	“Global CH_4 emissions from livestock increased from 31.8 [26.5–37.1] (mean [minimum−maximum of 95% confidence interval) Tg CH_4 yr−1 in 1890 to 131.7 [109.6–153.7] Tg CH_4 yr−1 in 2019, a fourfold increase in the past 130 years. The growth in global CH_4 emissions mostly occurred after 1950 and was mainly attributed to the cattle sector. Our estimate shows faster growth in livestock CH_4 emissions as compared to the previous Tier 1 estimates and is ~20% higher than the estimate from FAOSTAT for the year 2019.”
**22**	Auclair et al.	2021 [82]	Carbon footprint of Canadian self-selected diets	Canada	High-/low-GHG diets	DataFIELD (past LCA studies), survey	Carbon footprint	“High-GHGE diet respondents had a carbon footprint five-fold that of low-GHGE diet respondents (4.65 ± 0.07 vs. 0.90 ± 0.01 CO_2-eq per 1,000 kcal).”
**23**	Xu et al.	2021 [58]	Global GHGEs from animal-based foods are twice those of plant-based foods	Global	Plant- and meat-based food	After Poore & Nemecek (2018), LCA-based	GHGEs	“Global GHG emissions from the production of food were found to be 17,318 ± 1,675 $TgCO_2$eq yr−1, of which 57% corresponds to the production of animal-based food (including livestock feed), 29% to plant-based foods and 14% to other utilizations. Farmland management and land-use change represented major shares of total emissions (38% and 29%, respectively), whereas rice and beef were the largest contributing plant- and animal-based commodities (12% and 25%, respectively), and South and Southeast Asia and South America were the largest emitters of production-based GHGs.”
**24**	Errick-son et al.	2021 [41]	Animal-based foods have high social and climate costs	Global	General animal agricult-ure	Dynamic Integrated Climate–Economy model (incl.	GHGEs	“Under a business-as-usual (BAU) scenario, DICE-FARM projects that animal agriculture will account for 0.4 °C of the approximately 3 °C of warming at the end of the century … The temperature gap between the BAU scenario and a hypothetical reference scenario in which emissions from animal agriculture are immediately

						ERF, GLEAM - LCA)		set to zero ('Vegan') opens immediately and grows modestly throughout the twenty-first century ... This difference is consistent with the 12–18% livestock-related GHG emissions cited in past studies ... and indirectly illustrates the challenge of staying under a 2 °C temperature target without global dietary change."
25	Liu et al.	2021 [55]	Rethinking methane from animal agriculture	USA	Cattle	GWP100, GWP*	Methane emissions	"Using GWP*, the projected climate impacts show that CH_4 emissions from the U.S. cattle industry have not contributed additional warming since 1986. Calculations show that the California dairy industry will approach climate neutrality in the next ten years if CH_4 emissions can be reduced by 1% per year, with the possibility to induce cooling if there are further reductions of emissions."
26	Mert-ens et al.	2020 [35]	Potential impact of meat replacers on nutrient quality and GHGEs of diets	Europe	Meat vs meat replacers	SHARP Indicators database (ALCA, incl. consump-tion)	GHGEs	"[A] partial shift from meat to meat replacers can lead to a one-third reduction in diet-related GHGE."
27	Huan-Niemi et al.	2020 [42]	The impacts of dietary change	Finland	Meat vs reduction / elimin-ation	FoodMin dietary model (incl. LCA)	Consumer waste, CO_2 from domestic land use & food processing	"According to the FoodMin model calculations (Saarinen et al. 2019); the CO_2 emissions of a diet decrease as the share of animal-based products in the diet decrease. Compared with the current diet, CO_2 emissions can be decreased by 13% and 19% in the 'Meats cut to half' diet and 'Meats cut to one third' diet, respectively. The CO_2 emissions from the fish-rich diet can be decreased by 30% ... Shifting to the vegan diet would decrease CO_2 emissions by 37% from the current diet."
28	Escri-bano et al.	2020 [71]	Dairy sheep farms in semi-arid rangelands: a carbon footprint dilemma between intensification and land-based grazing	Spain	Dairy sheep	LCA (incl. C sequestra-tion)	Carbon footprint	"Greenhouse gas emissions vary from 1.77 to 4.09 Kg CO_2eq/kg of milk, where the lowest values correspond to the most intensive farms and the highest values to the most extensive and least productive farms. Enteric fermentation, followed by feeding, are the emissions with the greatest impact. Enteric fermentation reaches its maximum value (52.22 % of the total emissions) in the most extensive farms. Carbon sequestration varies between 0.09 and 2.04 kg of CO_2eq/kg of milk, a figure that can considerably reduce the carbon footprint calculation and justifies its inclusion in the Life Cycle Assessment."
29	Kok et al.	2020 [52]	European biodiversity assessments in livestock science	Europe	General animal agricult-ure	Review	Biodiversity assessment (>5 metrics)	"The general impact of (more intensive) livestock on biodiversity was classified as negative in 36% of studies, positive in 21% of studies, both positive and negative in 15% of studies, and not applicable or neutral in 26% of studies. Opposing patterns could be seen between food production and conservation functions. While studies in which livestock had a food

								production function found the impact of more intensive farming to be mostly negative, and never found it to be only positive, studies with a conservation function found the impact of livestock to be positive for biodiversity in almost half of the cases.”
**30**	Berar-dy et al.	2020 [60]	Environmental impacts of foods in the Adventist health study	Advent-ists in USA/ Canada	Meat, fish, animal products, plant	LCA (farm-to-gate & processing)	GWP, land use, water use	“Meats had the highest environmental impacts per both weight and protein content, while the lowest overall impacts per kilogram came from fruits. Meat analogs had the lowest overall impacts [GWP, land use, water use] per kilogram of protein [~13kg CO_2-eq/kg protein, 5m^2a/kg protein, 6m^3/kg protein, respectively], contrary to expectations that additional processing would result in higher environmental impacts when compared to whole plant-based foods. Dairy and eggs, in particular, had higher impacts [>120kg CO_2-eq/kg protein, ~78m^2a/kg protein, ~8m^3/kg protein, respectively] even when compared to meat on this basis due to having relatively lower protein content but similar input requirements and associated emissions.”
**31**	Blanco - Murcia et al.	2019 [43]	Sustainable diets and meat consumption reduction	Colom-bia	Cow, chicken, beans, lentils	Nutrition Ecology Framework, prior LCA-based work (incl. post gate)	Carbon & water footprints	“… the water footprint of beef produced in Colombia under a grazing system corresponds to 7545 m3/ton; poultry produced under an industrial system in Colombia corresponds to 3246 m3/ton of water; red beans produced in Colombia equals 2671 m3/ton of water; and the global average water footprint for lentils is 5874 m3/ton … the total carbon footprint of beef, not including LUC, was 31.7 kg of CO_2eq per kg of carcass weight, and including LUC was 47.7 kg CO_2eq per kg of carcass weight … The total carbon footprint of poultry in Colombia, including production and post-farm gate emissions, was estimated to be 6.9 kg of CO_2eq per kg of product … the emissions from the production of red beans corresponded to … a total carbon footprint of 2 kg of CO_2eq per kg of product … the total calculated carbon footprint for lentils was 1 kg of CO_2eq per kg of product.”
**32**	Adhik-ari et al.	2019 [36]	Environmental sustainability of food consumption	Asia: Thailand India China Japan Saudi Arabia	Grouped focus: meat, fish, animal products, plant	ALCA (main), CLCA (cradle-to-gate)	6 aspects	“The environmental impact categories chosen were global warming, terrestrial acidification, eutrophication, eco-toxicity, human toxicity, and fossil resource scarcity … For GWP, meat and cereals are the chief contributors in all the countries, with meat contributing to as high as 571 kg CO_2 eq (40%) in China. Meat consumption contributes to more than 25% in all countries except India for GWP. Cereals are the highest contributor to GWP in Thailand, contributing to 234 kg CO_2 eq (29% of total GWP) [*see paper for exhaustive figures*].”
**33**	Sintori et al.	2019 [47]	GHGEs in dairy goat farming systems	Greece	Dairy goats: extensive	Optimiz-ation model (linear	GHGEs	“The extensive farm causes higher emissions/kg of milk produced (4.08 kg CO_2-eq) compared to the semi-intensive and intensive farms (2.04 kg and 1.82 kg of CO_2-equivelants, respectively).”

					vs (semi)int-ensive	program-ming)		
34	Yau et al.	2018 [63]	Impact of cutting meat intake on hidden GHGEs in an import-reliant city	Hong Kong	Reducing meat	Hybrid LCA (incl. consump-tion), GLEAM	GHGEs	"Emissions solely from meat and dairy consumption were higher than the city's total greenhouse gas emissions using conventional production-based calculation ... government reports underestimate more than half of the emissions, as 62% of emissions are embodied in international trade. The discrepancy emphasizes the need of transitioning climate targets and policy to consumption-based accounting ... dietary change from a meat-heavy diet to a diet in accordance with governmental nutrition guidelines could achieve a 67% reduction in livestock-related emissions, allowing Hong Kong to achieve the Paris Agreement targets for 2030."
35	Poore et al.	2018 [27]	Reducing food's environmental impacts through producers and consumers	Global	40 meat and plant products	Meta-analysis of LCA studies, beyond farm gate?	Land use, freshwater, GHG, acidifying/ eutrophying emissions	"Impact can vary 50-fold among producers of the same product, creating substantial mitigation opportunities. Most strikingly, impacts of the lowest-impact animal products typically exceed those of vegetable substitutes, providing new evidence for the importance of dietary change."
36	Wei et al.	2018 [38]	Greenhouse gas and ammonia emissions and mitigation options from livestock production in peri-urban agriculture	China	Pig, dairy, beef cattle, poultry, sheep	Inventory generation (cradle-to-farm gate)	GHGs, ammonia	"Total industrial livestock production increased by 17% between 2010 and 2014. But lowered emissions due to legislation."
37	Arrieta et al.	2018 [50]	Impact of current, national dietary guidelines and alternative diets on GHGEs	Argen-tina	Current vs altern-ative diets	Prior LCI-based studies (excl. consump-tion)	GHGEs	"We found that the GHGE ... are very high (5.48 ± 1.71 kg CO_2-eq/person/day), with beef production contributing to the largest share of emissions (71%). The NDG suggest a 50% reduction of total daily intake of meats compared to current consumption, which ... would reduce GHGE in 28%, to 3.95 ± 0.96 ... The scenarios with non-ruminant meats and lacto-ovo vegetarian lead to similar GHGE, 2.11 ± 0.41 and 1.73 ± 0.37 kg CO_2-eq/day/person, respectively; and the vegan diet results in the lowest, 1.47 ± 0.34 kg CO_2-eq/day/person."
38	Bava et al.	2017 [59]	Environmental impact of the typical heavy pig production in Italy	Italy	Heavy pig vs standard	ALCA (cradle-to-gate), incl. imported feed land	9 aspects	"Production of heavy pigs generated environmental impacts per kg LW generally higher than the production of standard pigs slaughtered at lighter weight. The differences are particularly important for GWP [*see paper for exhaustive figures*]."

						use (change) and some post-gate		
39	White et al.	2017 [74]	Nutritional and greenhouse gas impacts of removing animals from US agriculture	USA	Removing meat	Prior LCA-based studies (some limits)	GHGs	"The modeled removal of animals from the US agricultural system resulted in predictions of a greater total production of food, increases in deficient essential nutrients and excess of energy in the US population's diet, a potential increase in foods/nutrients that can be exported to other countries, and a decrease of 2.6 percentage units in US GHG emissions."
40	Leip et al.	2015 [70]	Impacts of European livestock production	Europe	General animal agricult-ure	Extended CAPRI N-ALCA (cradle-to-gate, carbon seques-tration)	6 aspects	"Livestock sector contributes significantly to agricultural environmental impacts. This contribution is 78% for terrestrial biodiversity loss, 80% for soil acidification and air pollution (ammonia and nitrogen oxides emissions), 81% for global warming, and 73% for water pollution (both N and P). The agriculture sector itself is one of the major contributors to these environmental impacts, ranging between 12% for global warming and 59% for N water quality impact."
41	Tem-me et al.	2015 [62]	GHGE of diets in the Netherlands and associations with food, energy and macronutrient intakes	Nether-lands	Different diets	LCA, incl. some post-gate (type not specified)	GHGEs	"The GHGE of daily diets was on average 3·2 kg CO_2-equivalents (CO_2e) for girls, 3·6 kg CO_2e for boys, 3·7 kg CO_2e for women and 4·8 kg CO_2e for men. Meat and cheese contributed about 40 % and drinks (including milk and alcoholic drinks) 20 % to daily GHGE. Considerable differences in environmental loads of diets existed within age and gender groups. Persons with higher-GHGE diets consumed more (in quantity of foods and especially drinks) than their counterparts of a similar sex and age with low-GHGE diets. Major differences between high- and low-GHGE diets were in meat, cheese and dairy consumption as well as in ... drinks ... Of those, differences in meat consumption determined the differences in GHGE most."
42	West-hoek et al.	2014 [49]	Food choices, health and environment: effects of cutting Europe's meat and dairy intake	EU	Meat, dairy reduction	CAPRI, MITERRA-Europe, GAINS models	N, GHGs, land use	"Halving the consumption of meat, dairy products and eggs in the European Union would achieve a 40% reduction in nitrogen emissions, 25–40% reduction in greenhouse gas emissions and 23% per capita less use of cropland for food production. The European Union would become a net exporter of cereals, while the use of soymeal would be reduced by 75%. The nitrogen use efficiency (NUE) of the food system would increase from the current 18% to between 41% and 47% ... this is expected to result in a significant improvement in both air and water quality in the EU."

43	Head et al.	2014 [51]	Life cycle impacts of protein-rich foods	Nether-lands	Protein-rich foods (animal, non-animal)	LCI(A)	Biodiver-sity, climate change	“There are large differences between the product groups, but also within product groups. The main causes for the differences in biodiversity, climate change and land use change are as a result of the differences in livestock management, feed, feed conversion and greenhouse gas production by ruminants [*see paper for exhaustive figures*].”
44	De Carv-alho et al.	2013 [54]	Excessive meat consumption in Brazil	Brazil	Red/processed meat	Survey / Cedeberg et al. (2011) metrics: carbon footprint incl. land use (change)	CO_2 equivalents	“Mean red and processed meat intake was 138 g/d for men and 81 g/d for women. About 81% of men and 58% of women consumed more meat than recommended. In Brazil alone, GHGEs from meat consumption, in 2003, were estimated at approximately 18 071 988 tonnes of CO_2 equivalents, representing about 4% of the total CO_2 emitted by agriculture.”
45	Leht-onen et al.	2013 [48]	Impacts of reducing red meat consumption on agricultural production	Finland	Red meat (beef, pork, lamb)	Simulation, Dremfia model	Land use, GHGs	“Reduced red meat consumption would be offset by increases in consumption of poultry meat, eggs, dairy, and fish. Assuming unchanged consumer preferences, a 20% reduction is not likely to lead to a substantial reduction in land use or greenhouse emissions from Finnish agriculture.”
46	Cao et al.	2011 [61]	Life cycle assessment of Chinese shrimp farming systems targeted for export and domestic sales	China	Shrimp (intensive, export to Chicago; semi-intensive, domestic)	LCA, cradle-to-farm gate (main) / cradle-to-destination port	5 aspects	“In 2008, the estimated total electricity consumption, energy consumption, and greenhouse gas emissions from Chinese white-leg shrimp production would be 1.1 billion kW 3 h, 49 million GJ, and 4 million metric tons, respectively. Intensive farming had significantly higher environmental impacts per unit production than semi-intensive farming in all impact categories. The grow-out stage contributed between 96.4% and 99.6% of the cradle-to-farm-gate impacts. These impacts were mainly caused by feed production, electricity use, and farm-level effluents.”
47	Carew	2010 [45]	Ammonia emissions from livestock industries in Canada	Canada	General animal agricult-ure	Mass balance NH3 inventory, after Sommer (2003)	Ammonia emissions	“Emissions from animal agriculture accounted for 322 kilotonnes (kt) or 64% of Canadian NH3 emissions in 2002. Cattle and swine accounted for the bulk of livestock emissions. The provinces of Alberta, Ontario, Quebec, and Saskatchewan accounted for 28.1%, 22.0%, 18.7%, and 13.1% of total livestock emissions, respectively. Emissions from Ontario and Quebec were attributed to the intensive production of dairy, hogs and poultry. Dairy cattle emissions per hectolitre of milk were higher in Ontario and Québec than in other provinces, while swine emissions per livestock unit were higher than either beef or dairy cattle.”
